# A novel antifibrotic strategy utilizing conditioned media obtained from miR-150-transfected adipose-derived stem cells: validation of an animal model of liver fibrosis

**DOI:** 10.1038/s12276-020-0393-1

**Published:** 2020-03-09

**Authors:** Kwang Yeol Paik, Kee-Hwan Kim, Jung Hyun Park, Jae Im Lee, Ok-Hee Kim, Ha-Eun Hong, Haeyeon Seo, Ho Joong Choi, Joseph Ahn, Tae Yun Lee, Say-June Kim

**Affiliations:** 1 Department of Surgery, Yeouido St. Mary’s Hospital, College of Medicine, the Catholic University of Korea, Seoul, Republic of Korea; 2 Department of Surgery, Uijeongbu St. Mary’s Hospital, College of Medicine, the Catholic University of Korea, Seoul, Republic of Korea; 3 Catholic Central Laboratory of Surgery, Institute of Biomedical Industry, College of Medicine, the Catholic University of Korea, Seoul, Republic of Korea; 4 Department of Surgery, Eunpeong St. Mary’s Hospital, College of Medicine, the Catholic University of Korea, Seoul, Republic of Korea; 5 Department of Surgery, Seoul St. Mary’s Hospital, College of Medicine, the Catholic University of Korea, Seoul, Republic of Korea

**Keywords:** Adult stem cells, Experimental models of disease

## Abstract

The limitations of stem cells have led researchers to investigate the secretome, which is the secretory materials in stem cells, since the principal mechanism of action of stem cells is mediated by the secretome. In this study, we determined the antifibrotic potential of the secretome released from miR-150-transfected adipose-derived stromal cells (ASCs). The secretome released from ASCs that were transfected with antifibrotic miR-150 was obtained (referred to as the miR-150 secretome). To validate the antifibrotic effects of the miR-150 secretome, we generated in vitro and in vivo models of liver fibrosis by treating human hepatic stellate cells (LX2 cells) with thioacetamide (TAA) and subcutaneous injection of TAA into mice, respectively. In the in vitro model, more significant reductions in the expression of fibrosis-related markers, such as TGFβ, Col1A1, and α-SMA, were observed by using the miR-150 secretome than the control secretome, specifically in TAA-treated LX2 cells. In the in vivo model, infusion of the miR-150 secretome into mice with liver fibrosis abrogated the increase in serum levels of systemic inflammatory cytokines, such as IL-6 and TNF-α, and induced increased expression of antifibrotic, proliferation, and antioxidant activity markers in the liver. Our in vitro and in vivo experiments indicate that the miR-150 secretome is superior to the naive secretome in terms of ameliorating liver fibrosis, minimizing systemic inflammatory responses, and promoting antioxidant enzyme expression. Therefore, we conclude that miR-150 transfection into ASCs has the potential to induce the release of secretory materials with enhanced antifibrotic, proliferative, and antioxidant properties.

## Regenerative medicine: Training stem cells to fix liver fibrosis

A mixture of molecules produced by genetically modified stem cells could help repair the damage associated with liver fibrosis. Fat-derived adipose stem cells (ASCs) secrete proteins and nucleic acids that can facilitate tissue regeneration, but the natural mixture of molecules secreted (the ‘secretome’) is insufficient to reverse advanced fibrosis. Researchers led by Say-June Kim of the Catholic University of Korea, Seoul, South Korea, have boosted the potency of this cell-derived treatment by engineering ASCs to produce an RNA called miR-150. This RNA inhibits biological processes that drive fibrosis. Experiments in cultured cells and a mouse model of fibrosis confirmed that miR-150 consistently improved the ASC secretome’s capacity to control liver fibrosis and minimize systemic inflammatory responses. This approach could thus offer a safe strategy for promoting tissue regeneration and preventing liver failure.

## Introduction

Advanced liver fibrosis is one of the major causes of mortality and morbidity worldwide because it leads to liver cirrhosis, portal hypertension, hepatocellular carcinoma, and ultimately hepatic failure. Currently, liver transplantation is considered the only curative option. In the past few decades, advances in stem cell research have provided hope that stem cells could ameliorate liver fibrosis. However, despite several promising results in clinical trials^1–7^, it appears that it will take time for this approach to be applied in clinical practice. Safety concerns are one of the main obstacles to the clinical application of stem cells because the stability of transplanted stem cells cannot be guaranteed. To overcome safety concerns, several researchers have turned to their attention to the secretome, which are the molecules that are secreted or surface-shed by stem cells^8,9^. Numerous studies have demonstrated that the secretome has similar potential to that of stem cells because the principal mechanism of action of these cells is mediated by the secretome^10–17^. We have also performed several experiments that validated the therapeutic potential of the secretome, especially for the treatment of liver diseases, and have concluded that the secretome and stem cells have similar therapeutic potential, at least for improving hepatic failure^18–23^.

Although the secretome has some degree of regenerative properties, the naive secretome does not sufficiently improve advanced liver diseases. To improve the therapeutic potential of the stem cell-derived secretome, several modifications have been attempted, including alterations in the physicochemical culture environment and genetic modifications of stem cells^17,18,20,24^. There are advantages in utilizing antifibrotic microRNAs (miRNAs); miRNAs are small non-coding RNA molecules (~22 nucleotides) that alter gene expression at the posttranscriptional level, resulting in altered protein synthesis. miRNAs are involved in fibrosis, acting as profibrotic or antifibrotic miRNAs. Of these, miR-150 is a representative antifibrotic miRNA that inhibits the activation of hepatic stellate cells (HSCs)^25–31^. We hypothesized that transfection of miR-150 into adipose-derived stem cells (ASCs) will promote the release of a secretome with robust antifibrotic properties. Accordingly, we generated a secretome from miR-150-transfected ASCs (miR-150 secretome) and determined its antifibrotic properties using both in vitro and in vivo experimental models of liver fibrosis.

## Materials and methods

### Preparation of cells

LX-2 human stellate cells were kindly donated by Dr. Won-il Jeong of KAIST Biomedical Research of Korea. LX-2 cells were maintained in DMEM (Dulbecco’s modified Eagle’s medium; Thermo Fisher Scientific, Carlsbad, CA). The medium was supplemented with 10% FBS (fetal bovine serum; Gibco BRL, Carlsbad, CA) and 1% antibiotics (Thermo Fisher Scientific) at 37 °C.

Human adipose-derived stromal cells (ASCs) were obtained from lipoaspirated fat with informed consent from the volunteers. This research was approved by the Institutional Review Board (IRB number: 700069-201407-BR-002-01) of Hurim BioCell Co. Ltd. (Seoul, Korea). Lipoaspirated fat was digested by 0.1% collagenase (Sigma-Aldrich, St. Louis, MO) in saline and collected after centrifugation. The cells were plated in culture flasks in low-glucose DMEM supplemented with 10% FBS (Thermo Fisher Scientific), 100 U/mL penicillin (Thermo Fisher Scientific), and 0.1 mg/mL streptomycin (Thermo Fisher Scientific). ASCs were incubated at 37 °C in a humidified chamber containing 5% carbon dioxide, and the medium was changed every 3 days.

### miR-150 transfection into ASCs

ASCs were transfected with miRNA-150 (miR-150; Exiqon, Germantown, MD) using Lipofectamine RNAiMAX reagent (Thermo Fisher Scientific) in medium. After 72 h of transfection, the cells were morphologically observed by inverted microscopy. The cell numbers were counted with an automatic cell counter (Countess; Invitrogen, San Diego, CA) using trypan blue solution. Transfected cells were processed for cell phenotyping or were differentiated with three-lineage induction.

For cell characterization, the immunophenotype of naive or transfected ASCs was determined by flow cytometry analysis (Cytomics FC500 flow cytometer, Beckman Coulter, Fullerton, CA) using FITC-conjugated CD31, CD45, and CD73 antibodies and PE-CD105 antibodies (BD Pharmingen, San Jose, CA). Isotype controls were performed with antibodies against IgG. In addition, the adipogenic, osteogenic, and chondrogenic differentiation ability of the naive and transfected ASCs was determined as previously described^32,33^.

### miRNA extraction and real-time PCR

Total miRNAs were prepared from ASCs and exosomes using a miRNeasy mini kit (Qiagen, Hilden, Germany). Total exosomes were prepared from ASC culture supernatant using total exosome isolation reagent (Thermo Fisher Scientific). RNA (250 ng) was reverse transcribed using a miRCURY LNA Universal RT microRNA PCR kit (Qiagen). Real-time PCR was performed using miR-150-specific primers (Qiagen). qRT-PCR was performed on a Step One Plus real-time PCR system (Life Technologies, Carlsbad, CA). After normalization to U6 snRNA (Qiagen), expression levels of each target miRNA were calculated using the comparative cycle threshold method.

### Western blot analysis

LX-2 cells and liver specimens were obtained from mice, lysed using a EzRIPA lysis kit (ATTO Corporation; Tokyo, Japan) and quantified by Bradford reagent (Bio-Rad, Hercules, CA). Proteins were visualized by western blot analysis using primary antibodies (1:1000 dilution) at 4 °C overnight and then HRP-conjugated secondary antibodies (1:2000 dilution) for 1 h at 25 °C. Primary antibodies against PCNA (proliferating cell nuclear antigen), TGF-β1 (transforming growth factor-β1), α-SMA (alpha-smooth muscle actin), TIMP-1 (metallopeptidase inhibitor 1), MMP2 (matrix metallopeptidase 2), COl1A1 (collagen type I alpha 1 chain), and β-actin and horseradish peroxidase (HRP)-conjugated secondary antibodies were obtained from Cell Signaling Technology (Beverly, MA). Specific immune complexes were detected using Western Blotting Plus chemiluminescence reagent (Millipore, Bedford, MA).

### Animal study using experimental mice

We used 5-week-old male BALB/c mice (Orient Bio, Seongnam, Korea) in this study. Animal studies were carried out in compliance with the guidelines of the Institute for Laboratory Animal Research of Korea (IRB No: CUMC-2018-0175-01). We then compared the effects of MCM in an in vivo model of TAA-induced hepatic fibrosis. The in vivo model was generated by subcutaneous injection of TAA (200 mg/kg, three times a week for 5 weeks) into the mice. The mice were intravenously infused with normal saline (0.1 mL of saline; *n* = 12), the control secretome (0.1 mL equivalent of the 25-fold concentrated secretome obtained from 10^5^ ASCs after 24 h culture in serum-free medium, *n* = 12), or the miR-150 secretome (0.1 mL equivalent of the 25-fold concentrated serum-free medium obtained from 10^5^ ASCs that were transfected with miR-150, *n* = 12) two times a week for 1 week.

### ELISA

Blood samples were collected from each mouse and centrifuged for 10 min at 9500 × *g*, and serum was collected. The concentrations of mouse interleukin (IL)-6 and tumor necrosis factor (TNF)-α were measured by sandwich enzyme-linked immunosorbent assay (ELISA) (Biolegend, San Diego, CA) according to the manufacturer’s instructions.

### Specialized and immunohistochemical stains

Masson’s trichrome staining and Sirius red staining were performed using the respective staining kits (Polysciences, Warrington, PA) according to the manufacturer’s protocol.

For immunohistochemical analysis, formalin-fixed, paraffin-embedded tissue sections were deparaffinized, rehydrated in an ethanol series and subjected to epitope retrieval using standard procedures. Antibodies against albumin, α-SMA, and superoxide dismutase (SOD) (all from Cell Signaling Technology, MA) were used for immunochemical staining. The samples were then examined under a laser-scanning microscope (Eclipse TE300; Nikon, Tokyo, Japan) to analyze the expression of these proteins.

### Statistical analysis

All data were analyzed using a standard software package (SPSS 11.0; SPSS Inc., Chicago, IL). All data are reported as the means ± standard deviations. Statistical comparison among groups was determined using the Kruskal–Wallis test. Probability values of *P* < 0.05 were regarded as statistically significant.

## Results

### Effects of miR-150 transfection on the functionality of ASCs

**Fig. 1:**
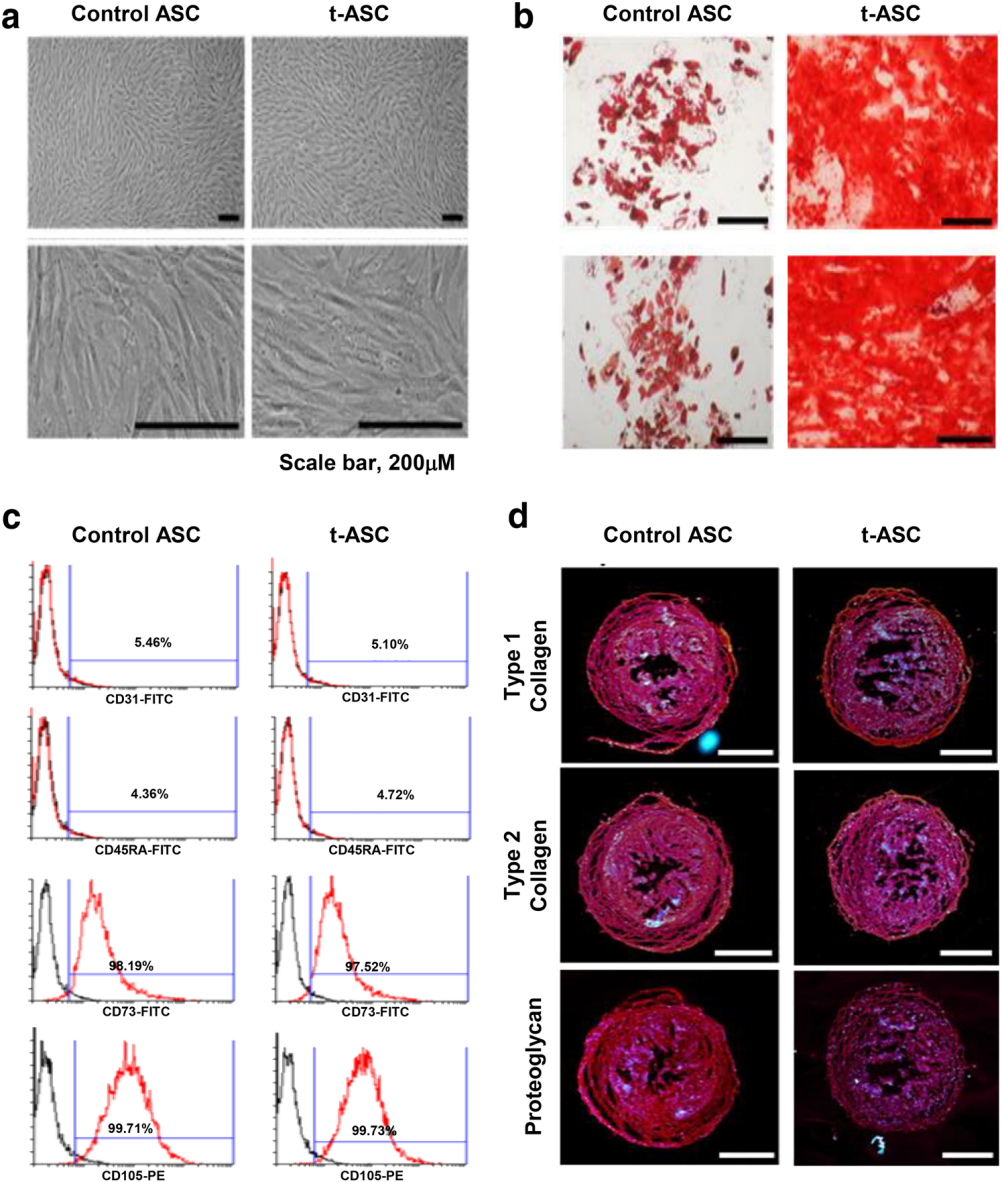
Assessment of the multilineage differentiation potential of miR-150-transfected ASCs. **a** Comparison of gross cell morphology of ASCs with/without miR-150 transfection. There was no difference in gross cell morphology between the two groups. **b** Flow cytometric analysis of ASCs transfected with miR-150. The miR-150-transfected ASCs were negative for CD31 and CD45 (hematopoietic stem cell markers) and positive for CD73 and CD105 (mesenchymal stem cell markers), and the expression pattern in ASCs without miR-150 transfection was identical to that of transfected ASCs. **c** Successful adipogenic [Top] and osteogenic [Bottom] differentiation of miR-150-transfected ASCs. Adipogenic and osteogenic differentiation were identified using Oil Red O and Alizarin red stains, respectively. **d** Successful chondrogenic differentiation of miR-150-transfected ASCs. Chondrogenic differentiation was identified using collagen types 1 and 2 and proteoglycan stains. Scale bars = 100 µm. The values are presented as the mean ± standard deviation of three independent experiments. *P < 0.05. ASC adipose-derived stem cell, t-ASC miR-150-transfected adipose tissue-derived stem cell.

We investigated whether miR-150 transfection impacted the functionality of ASCs, including surface marker expression and multilineage differentiation potential. MiR-150 transfection did not affect the gross morphology of cultured ASCs (Fig. 1a). MiR-150 transfection did not alter the expression of ASC surface markers, as determined by flow cytometry (Fig. 1b). MiR-150 transfection also did not impair the multilineage differentiation potential of ASCs, as demonstrated by the successful differentiation of miR-150-transfected ASCs into adipogenic, osteogenic, and chondrogenic cells (Fig. 1c, d).

### Antifibrotic effects of the miR-150 secretome in an in vitro model of liver fibrosis

**Fig. 2:**
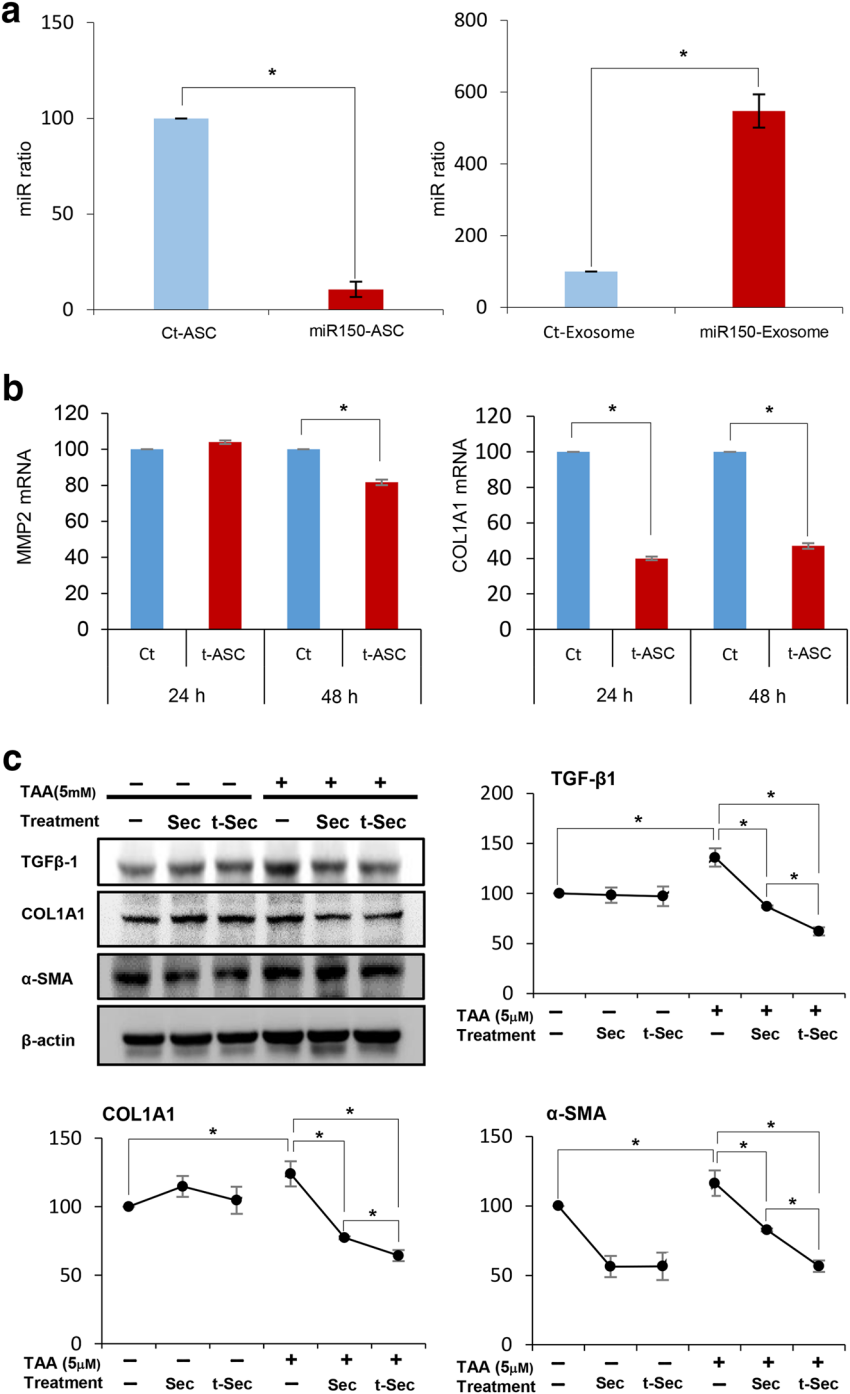
Antifibrotic effects of the miR-150 secretome in an in vitro model of liver fibrosis. We transfected antifibrotic miR-150 into ASCs and determined the expression of fibrosis-related markers in the transfected ASCs. **a** Real-time PCR analysis to determine **miR-150** expression in miR-150-transfected ASCs at 24 h posttransfection. At 24 h posttransfection, decreased miR-150 expression in miR-150-transfected ASCs and increased miR-150 expression in the exosomes of miR-150-transfected ASCs were observed, suggesting that miR-150 was released and accumulated in exosomes at 24 h after transfection. **b** RT-PCR results showing the mRNA expression of *MMP2* and *COL1A1* in miR-150-transfected ASCs. MiR-150-transfected ASCs expressed significantly lower mRNA levels of *MMP2* and *COL1A1* than control ASCs, particularly at 48 h after transfection. **c** Western blot analysis of the effects of the miR-150 secretome on the expression of fibrosis-related markers in an in vitro model of liver fibrosis. The in vitro model of liver fibrosis was established by treating human stellate cells (LX2 cells) with the hepatotoxin thioacetamide (TAA) (2.5 mM). The miR-150 secretome reduced the expression of fibrosis-related markers, such as TGFβ, Col1A1, and α-SMA, more significantly than that of the control secretome, particularly in the in vitro model of liver fibrosis. The values are presented as the mean ± standard deviation of three independent experiments. *P < 0.05. α-SMA alpha-smooth muscle actin, COL1A1 collagen type I alpha 1 chain, Ct control, MMP2 metalloproteinases-2, Sec the secretome obtained from ASCs after 48 h of incubation, TAA thioacetamide, t-ASC miR-150-transfected adipose-tissue derived stem cell, TGF-β transforming growth factor-β, TIMP-1 tissue inhibitor of metalloproteinases-1, t-Sec the secretome released from miR-150-transfected ASCs.

To clarify the posttransfection expression of miR-150 in ASCs, we measured the expression of miR-150 using real-time PCR after extracting the miRNAs from ASCs 24 h and 48 h after transfection. At 24 h posttransfection, decreased miR-150 expression was observed in miR-150-transfected ASCs, and increased miR-150 expression was observed in the exosomes of miR-150-transfected ASCs (*P* < 0.05) (Fig. 2a). However, at 48-h posttransfection, there was no miRNA expression in either miR-150-transfected ASCs or their exosomes (data not shown). These results suggest that miR-150 accumulated and was released in exosomes 24 h after transfection. In this experiment, the secretome obtained at 24 h after transfection was used; thus, miR-150 was expected to be present in the miR-150 secretome.

We transfected antifibrotic miR-150 into ASCs and determined the expression of fibrosis-related markers in the transfected cells. MiR-150-transfected ASCs expressed significantly lower levels of *MMP2* mRNA than control ASCs, particularly 48 h after transfection (*P* < 0.05) (Fig. 2b). Additionally, miR-150-transfected ASCs expressed significantly lower levels of *COL1A1* mRNA than control ASCs (*P* < 0.05).

Next, the miR-150 secretome was obtained from the conditioned media of miR-150-transfected ASCs after several purification steps that were described in the Methods section. We then established an in vitro model of liver fibrosis by treating human stellate cells (LX2 cells) with the hepatotoxin thioacetamide (TAA) (2.5 mM). The TAA-treated HSCs exhibited increased expression levels of fibrosis-related markers, such as TGFβ, Col1A1, and α-SMA, than those of control HSCs. Subsequently, we added either the control or miR-150 secretome to either the control or TAA-treated HSCs and determined the expression of fibrosis-related markers. The miR-150 secretome resulted in a more significant reduction in the expression of these markers than that of the control secretome, particularly in TAA-treated HSCs (*P* < 0.05) (Fig. 2c).

### Antifibrotic effects of the miR-150 secretome in an in vivo model of liver fibrosis

**Fig. 3:**
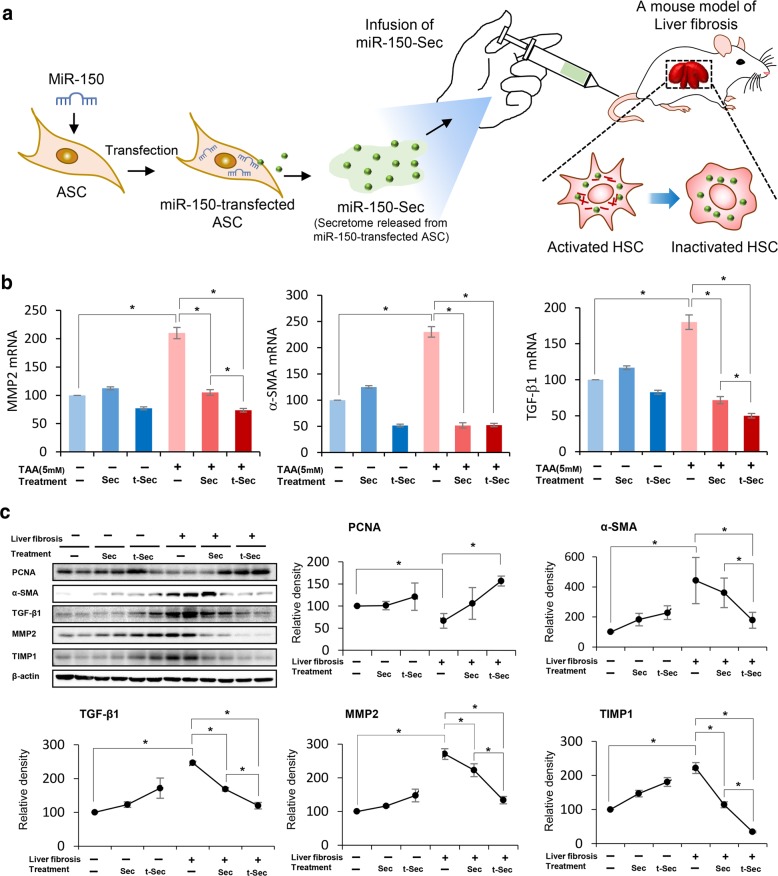
Antifibrotic effects of the miR-150 secretome in an in vivo model of liver fibrosis. **a** A schematic illustration showing the process of in vivo experiments. Mice with liver fibrosis were intravenously infused with normal saline (*n* = 12), the control secretome (*n* = 12), or the miR-150 secretome (*n* = 12) three times a week for 1 week. **b** RT-PCR results showing the mRNA expression of *MMP2*, *α-SMA*, and *TGF-β1* in liver specimens in each group. The infusion of either the control or miR-150 secretome significantly reduced the mRNA expression levels of *MMP-2, α-SMA*, and *TGF-β1* in the liver compared to those of infusion of normal saline, particularly in the mice with liver fibrosis. Comparing the two secretome groups, infusion of the miR-150 secretome induced significantly lower levels of *MMP-2* and *TGF-β1* expression in the liver specimens compared to those of infusion of the control secretome (*P* < 0.05). There was no significant difference in the expression of *α-SMA* between the two secretome groups. **c** Western blot analysis showing the effects of the miR-150 secretome on the expression of fibrosis-related markers in an in vivo model of liver fibrosis. Infusion of the miR-150 secretome induced higher expression of PCNA (a proliferation marker) and lower expression of fibrosis-related markers (α-SMA, TGF-β1, MMP-2, and TIMP-1) in the liver specimens compared with the levels obtained from infusion of the control secretome. The values are presented as the mean ± standard deviation of three independent experiments. *P < 0.05. α-SMA alpha-smooth muscle actin, Ct control, MMP-2 metalloproteinases-2, PCNA proliferating cell nuclear antigen, Sec the secretome obtained from ASCs after 48 h of incubation, TAA thioacetamide, TGF-β transforming growth factor-β; TIMP-1 tissue inhibitor of metalloproteinases-1, t-Sec the secretome released from miR-150-transfected ASCs.

To validate the effects of the miR-150 secretome in vivo, we established a mouse model of liver fibrosis by subcutaneous injection of TAA (200 mg/kg) three times a week for 5 weeks. The mice were intravenously infused with normal saline (*n* = 12), the control secretome (*n* = 12), or the miR-150 secretome (*n* = 12) two times a week for 1 week (Fig. 3a). Serum samples were collected on the determined days; the mice were euthanized, and liver specimens were obtained on the 7th day.

RT-PCR revealed that infusion of either the control or miR-150 secretome significantly reduced the mRNA expression levels of *MMP-2, α-SMA*, and *TGF-β1* in the liver compared to those of infusion of normal saline, particularly in mice with liver fibrosis (Fig. 3b) (*P* < 0.05). In comparing the two secretome groups, the miR-150 secretome group had significantly lower mRNA expression levels of *MMP-2* and *TGF-β* in the liver compared to those of the control secretome group (*P* < 0.05). There was no significant difference in the expression of *α-SMA* between the two secretome groups.

We then compared the expression of various proteins in each group by western blot analysis (Fig. 3c). The infusion of the miR-150 secretome resulted in increased expression levels of PCNA, a proliferation marker, compared to the levels obtained by infusion of the control secretome (*P* < 0.05). Additionally, lower levels of fibrosis-related markers, such as α-SMA, TGF-β1, MMP-2, and TIMP-1, were observed in liver specimens after infusion of the miR-150 secretome compared to those of infusion with the control secretome (*P* < 0.05).

**Fig. 4:**
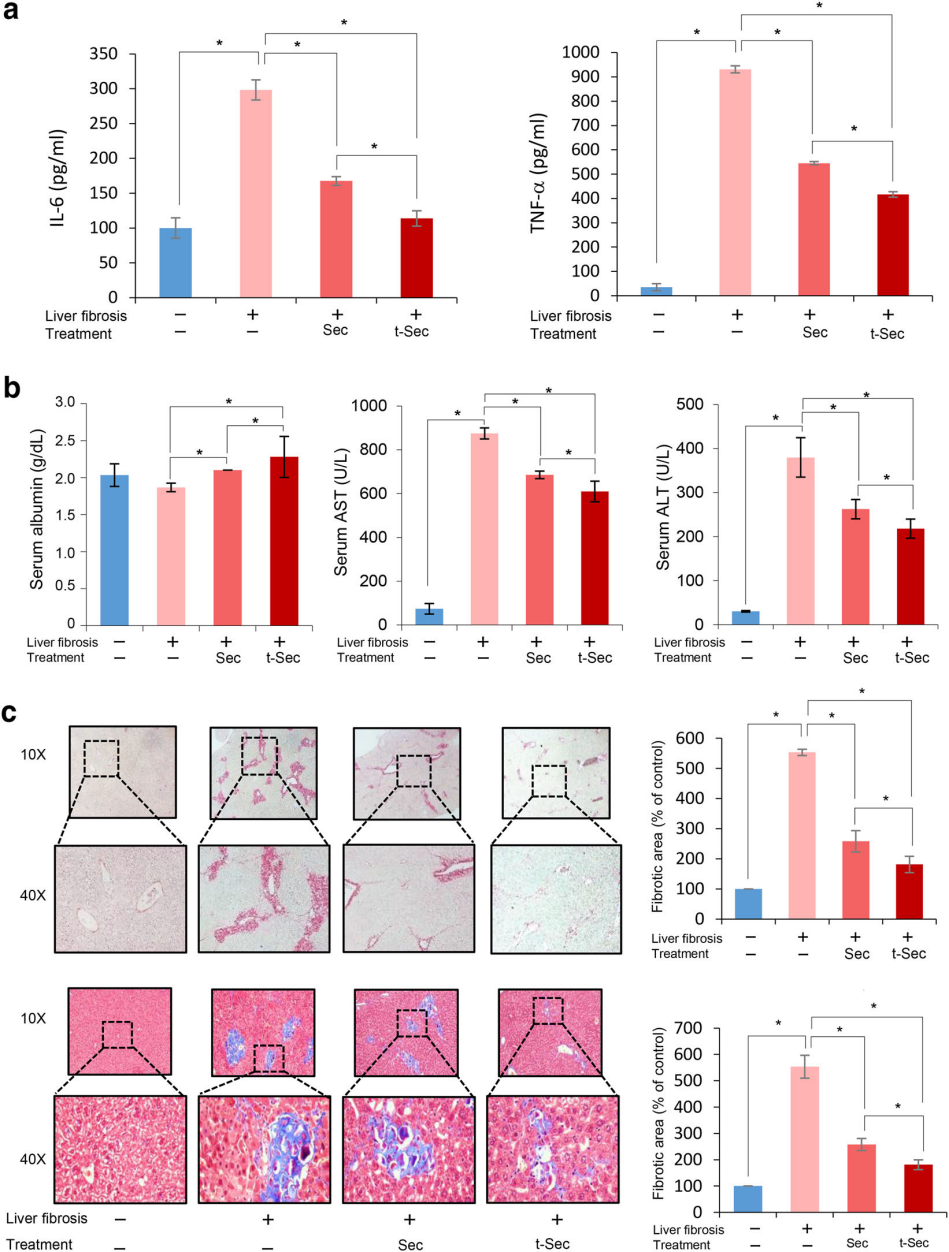
Determination of systemic inflammation and a histological assessment of liver specimens. **a** ELISA results demonstrating the serum levels of proinflammatory mediators, including IL-6 and TNF-α, in each group. Secretome infusions significantly reduced the serum levels of IL-6 and TNF-α in mice with liver fibrosis. When comparing both groups, miR-150 secretome infusion induced more significant reductions in these cytokines than those of the control secretome infusion group. **b** Serum levels of albumin, AST, and ALT in each group. The serum albumin concentration was highest in the miR-150 secretome group, followed by the control secretome group. Of the TAA-treated groups, the miR-150 secretome group showed the lowest levels of these liver enzymes, followed by the control secretome group. **c** Sirius red [Top left] and Masson trichrome [Bottom left] staining showing the degree of fibrosis. Both stains demonstrated that infusion of the miR-150 secretome decreased liver fibrosis more than that of infusion of the control secretome. [Right] Comparison of fibrotic areas in each group. Percentages of fibrotic areas were measured using NIH ImageJ and are expressed as relative values to those in normal livers. The values are presented as the mean ± standard deviation of three independent experiments. *P < 0.05. ALT alanine transaminase, AST aspartate transaminase, Sec the secretome obtained from ASCs after 48 h of incubation, TNF-α tumor necrosis factor-α, t-Sec the secretome released from miR-150-transfected ASCs.

Subsequently, we compared the serum levels of proinflammatory cytokines, including IL-6 and TNF-α, among the groups (Fig. 4a). Secretome infusions significantly reduced the serum levels of IL-6 and TNF-α, and the reductions in cytokine levels were more significant after infusion of the the miR-150 secretome than the control secretome (*P* < 0.05). Subsequently, we compared the serum levels of albumin, aspartate transaminase (AST), and alanine transaminase (ALT) in each group (Fig. 4b). The serum albumin concentration was highest in the miR-150 secretome group, followed by the control secretome group (*P* < 0.05). The serum levels of liver enzymes (AST and ALT) were significantly increased in the TAA group. Of them, the miR-150 secretome group showed the lowest levels of these liver enzymes, followed by the control secretome group (*P* < 0.05). We next performed Sirius red and Masson trichrome staining to determine the degree of liver fibrosis (Fig. 4c). Both stains demonstrated the antifibrotic effects of secretome infusion; moreover, the miR-150 secretome group had significantly greater improvement in fibrosis than that of the control secretome group (*P* < 0.05).

### Immunohistochemistry and liquid chromatography-mass spectrometry analysis of secretome components

**Fig. 5:**
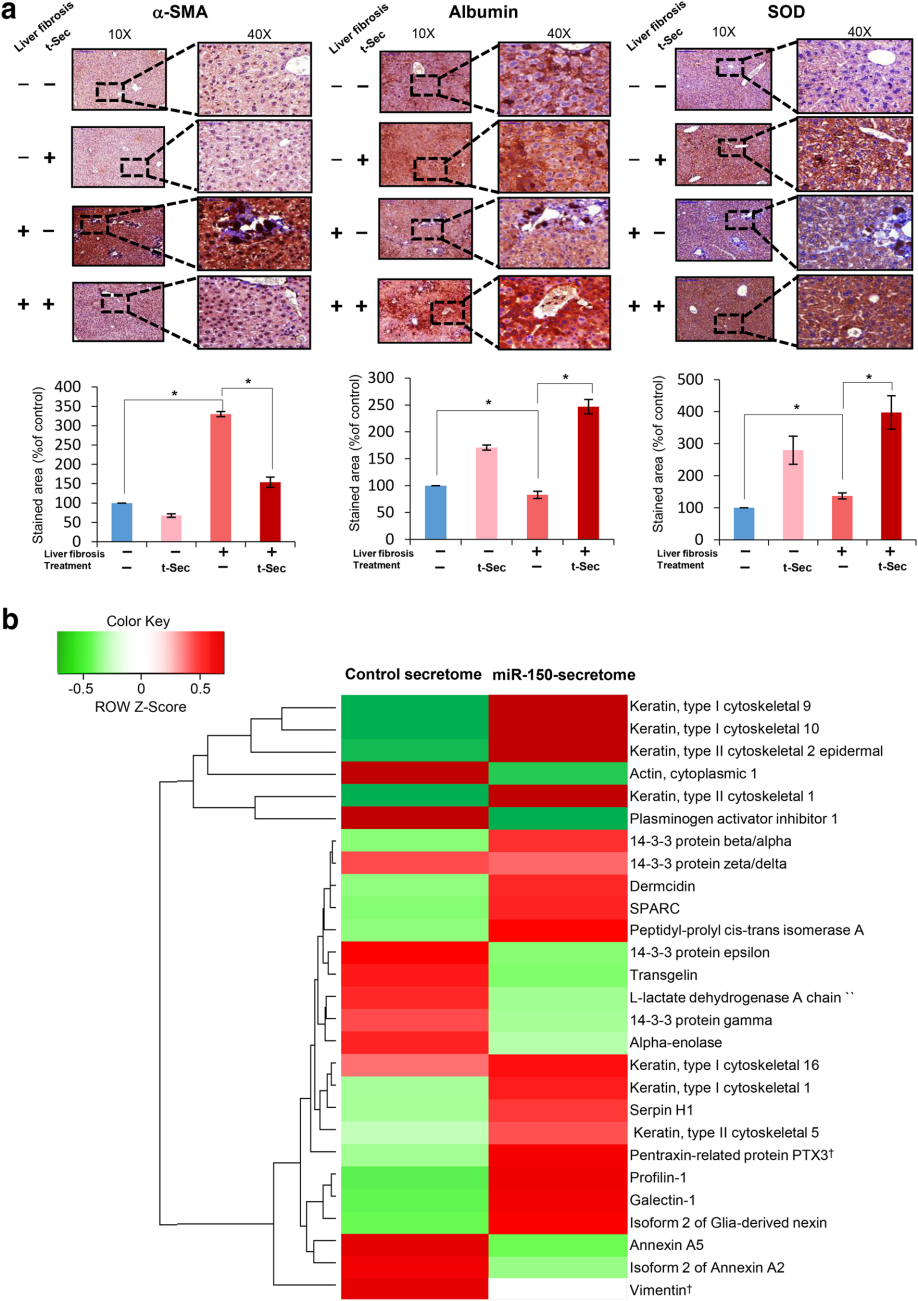
Immunohistochemistry and liquid chromatography-mass spectrometry analysis of secretome components. **a** α-SMA [Top left], albumin [Top middle], and SOD [Top right] Immunohistochemistry (IHC) analysis of livers in each group. [Bottom] Comparison of immunoreactive areas in each group. Percentages of immunoreactive areas were measured using NIH ImageJ and are expressed as relative values to those in normal livers. *P < 0.05. The values are presented as the mean ± standard deviation of three independent experiments. **b** Heat map generated from label-free liquid chromatography-mass spectrometry (LC-MS) showing quantitative proteomics reflecting the protein expression values of the control and miR-150-transfected secretomes. Samples are arranged in columns, and proteins are in rows. Red shades indicate increased expression in samples compared to that of control samples; green shades indicate reduced expression; black indicates median expression. Each LC-MS sample was pooled from two samples of the secretome. The components and concentrations of various essential proteins varied widely between the control and miR-150-transfected secretomes, validating their specificity. For instance, the miR-150 secretome contained a considerably reduced level of vimentin (a profibrogenic protein) and considerably increased expression of PTX3 (an antifibrogenic protein) compared to those of the control secretome. †Most prominent difference in protein concentration between the control secretome and miR-150 secretome. α-SMA alpha smooth muscle actin, PTX3 pentraxin-related protein 3, Sec the secretome obtained from ASCs after 48 h of incubation, t-Sec the secretome released from miR-150-transfected ASCs, SOD superoxide dismutase.

To determine the effects of the miR-150 secretome on the fibrotic liver, we used immunohistochemistry (IHC) to evaluate liver specimens. Antibodies against α-SMA, albumin, and SOD were used to determine the effects of the miR-150 secretome on liver fibrosis, hepatic synthesis function, and antioxidant activity, respectively. The fibrotic livers were characterized by increased levels of α-SMA and reduced levels of albumin and SOD (Fig. 5a). Based on α-SMA IHC results, infusion of the miR-150 secretome significantly reduced the expression of α-SMA in both fibrotic and normal livers. Based on albumin IHC results, infusion of the miR-150 secretome significantly increased the expression of albumin in both normal and fibrotic livers. Finally, based on SOD IHC results, infusion of the miR-150 secretome significantly decreased the expression of SOD in both normal and fibrotic livers. Taken together, the miR-150 secretome has the potential to enhance hepatic synthesis functions and antioxidant activity, as well as to reduce liver fibrosis.

Finally, liquid chromatography-mass spectrometry (LC-MS) was used to determine the components in the conditioned medium. The protein components in the control secretome were compared with those in the miR-150 secretome. LC/MS analysis of both sets of components detected many differences in protein expression in both groups (Fig. 5b). For example, the miR-150 secretome contained a considerably lower level of vimentin (a profibrogenic protein) and had considerably higher expression of PTX3 (an antifibrogenic protein) compared to those of the control secretome.

## Discussion

This study validated the antifibrotic and anti-inflammatory effects of the secretome released from miR-150-transfected ASCs. Our in vitro and in vivo experiments consistently demonstrated that miR-150 transfection improved the antifibrotic and anti-inflammatory properties of ASCs. In particular, intravenous infusion of the miR-150 secretome in mice with liver fibrosis resulted in (1) abrogation of the elevated systemic inflammatory cytokines, such as IL-6 and TNF-α, (2) a significant reduction in liver fibrosis, and (3) increased expression of markers of liver cell proliferation and liver antioxidant activities. Taken together, these findings suggest that miR-150 transfection in ASCs has the potential to induce the release of a secretome with improved antifibrotic, proliferative, and antioxidant effects than that of a naïve secretome.

MiR-150 is a representative antifibrotic miRNA. A recent investigation revealed several mechanisms by which miR-150 exerts antifibrotic effects. First, miR-150 inhibits HSC activation by inhibiting c-myb expression^29^. C-myb is a protooncogene that encodes a transcription factor that is involved in the proliferation, differentiation, and survival of hematopoietic cells^34^. C-myb is also expressed by activated HSCs and has a substantial role in the development of fibrosis^35^. In addition, c-myb has an established role in oxidative stress-induced production of type I collagen by activated HSCs^36^. Therefore, inhibiting c-myb by miR-150 exerts antifibrotic effects. Additionally, miR-150 reduces type I and IV collagen by directly binding to Sp1 and COL4A4, respectively, without the involvement of factors upstream of the TGF-β/Smad pathway^31^. Specifically, miR-150 targets the 3′ UTR region of *Sp1*. The interaction between miR-150 and the target regions in *Sp1* leads to a reduction in COL1A1, which is responsible for producing type I collagen. MiR-150 also interacts with the 3′ UTR of *COL4A4*, which encodes type IV collagen in fibrotic livers. Taken together, miR-150 is an antifibrogenic miRNA that not only inhibits the activation of HSCs but also reduces the production of type I and type IV collagen by HSCs.

Overall, treatment with the miR-150 secretome reduced the expression of profibrogenic markers, such as α-SMA, MMP2, and TGF-β, more than that of the control secretome in both in vitro and in vivo models of liver fibrosis. These changes were observed at both the RNA and protein levels, except for α-SMA. While the mRNA expression of α-SMA was similarly decreased in both the control and miR-150 secretome groups, the protein expression of α-SMA was significantly lower in the miR-150 secretome group than in the control secretome group in both in vitro and in vivo experiments due to unknown reasons. Further study is needed to determine the precise cause of this discrepancy.

In this study, we transfected ASCs but not HSCs with miR-150. We hypothesized that miR-150 has the potential to reprogram ASCs to release materials with improved antifibrotic properties. The release of miR-150 from miR-150-transfected ASCs is correlated with exosomes. At 24-h posttransfection, decreased miR-150 expression in miR-150-transfected ASCs and increased miR-150 expression in the exosomes of miR-150-transfected ASCs were observed. These results suggest that miR-150 accumulated and was released in exosomes 24 h after transfection. The miR-150 secretome was thus obtained at 24 h of transfection to include these kinds of exosomes. Intravenous infusion of the miR-150 secretome significantly reduced the expression of TGF-β1 in the livers of mice with liver fibrosis, suggesting that the miR-150 secretome has the potential to inhibit TGF-β1 signaling.

TGF-β/Smad signaling is an attractive therapeutic target for the treatment of liver fibrosis. Several anti-TGF-β strategies are currently available, including the administration of anti-TGF-β-neutralizing antibodies, soluble TβRs, and small molecule TβR kinase inhibitors^37^. We think that the miR-150 secretome has the potential to become a prominent anti-TGF-β strategy. TGF-β is the most potent factor that accelerates liver fibrosis in a number of ways, including by promoting HSC activation, stimulating collagen gene transcription, and inhibiting MMP expression^38^. Under the inflammatory microenvironment of the liver, TGF-β is secreted not only by platelets and Kupffer cells but also by activated HSCs themselves^39^. TGF-β is also highly expressed in activated HSCs. TGF-β first binds to the type II TGF-β receptor on the surface of HSCs. It recruits and phosphorylates the type I receptor, which in turn phosphorylates Smad2 and Smad3. TGF-β then forms a complex with Smad4, which is translocated from the cytoplasm to the nucleus, subsequently stimulating the transcription of genes that promote fibrogenesis. Our results suggest that interfering with TGF-β/Smad signaling is the principal mechanism of action of the miR-150 secretome.

There were considerable differences in the protein components of the control and miR-150 secretomes. Certain protein components were increased and the others were not changed or decreased in the miR-150 secretome compared to those of the control secretome. It is thought that the total number of these changes contributes to the robust antifibrogenic effect of the miR-150 secretome. Specifically, the miR-150 secretome contained a considerably lower levels of vimentin (a profibrogenic protein) and considerably higher expression of PTX3 (an antifibrogenic protein) compared to those of the control secretome. Vimentin is an essential element of inflammation and fibrosis. Walker et al.^40^ showed that vimentin mediates the transition of mesenchymal leader cells to a myofibroblast phenotype, facilitating fibrosis in wound repair, while vimentin deficiency resulted in a blunted inflammatory response. Vimentin is also a key regulator of the NLRP3 inflammasome, promoting lung inflammation and fibrosis^13^. In addition, PTX3 inhibits inflammation and leukocyte adhesion and transmigration^41^. PTX3 produced by monocytes/macrophages abolishes neutrophil recruitment and oxidative bursts^42^. PTX3 also cooperates with the adaptive immune system in defense against infectious agents by developing a protective Th1/Treg immune response^43^. Thus, identifying the roles of the altered protein components in the miR-150 secretome is expected to help identify antifibrogenic materials in the future.

In conclusion, our in vitro and in vivo experiments indicate that the miR-150 secretome is superior to the naïve secretome in terms of ameliorating liver fibrosis, minimizing systemic inflammatory responses, and promoting antioxidant activities of the liver. Accordingly, we think that miR-150 transfection has the potential to induce the release of secretory materials from ASCs that have enhanced antifibrotic, proliferative, and antioxidant properties. We also showed that interfering with TGF-β/Smad signaling may be the principal mechanism of action of the miR-150 secretome. We thus conclude that the miR-150 secretome is a potential anti-TGF-β strategy against liver fibrosis.
